# Older People’s Usage Pattern, Satisfaction with Community Facility and Well-Being in Urban Old Districts

**DOI:** 10.3390/ijerph191610297

**Published:** 2022-08-18

**Authors:** Siqiang Wang, Esther Hiu Kwan Yung, Ester Cerin, Yifan Yu, Peiheng Yu

**Affiliations:** 1 Department of Building and Real Estate, The Hong Kong Polytechnic University, Hong Kong, China; 2 Mary MacKillop Institute for Health Research, Australian Catholic University, Melbourne, VIC 3000, Australia; 3 School of Public Health, The University of Hong Kong, Hong Kong, China; 4 College of Architecture and Urban Planning, Tongji University, Shanghai 200092, China

**Keywords:** community facility, usage pattern, well-being, older people, urban old district

## Abstract

Community facilities are an important element that supports older people’s daily life and promotes their well-being. However, there is a dearth of comprehensive studies on the effect of planning and design of different types of community facilities on older people’s usage patterns and satisfaction. This study aims to provide a framework to explore the relationship among the planning of community facilities, older people’s usage and satisfaction level and well-being for different types of community facilities. Both spatial analysis and questionnaire survey (*n* = 497) methods are employed in this study. This study finds that commercial (89.34%), municipal (83.10%) and leisure (88.13%) facilities are most commonly used by older people. This study suggests that older people’s frequency of visiting community facilities is mainly affected by the purpose of visiting a community facility. Planning and design quality of the community facility are found to be significantly associated with older people’s satisfaction level with using a community facility. In addition, older people’s higher satisfaction level and usage level of community facilities could increase their physical and psychological well-being. The findings of this study not only contribute to the knowledge gap of older people’s usage and satisfaction with using community facilities but also suggest that planners should aim toward a better distribution of community facilities to improve older adults’ well-being.

## 1. Introduction

Under the trend of rapidly aging population, the promotion of active aging and aging in place has become significantly important [1]. It has been seen as a popular solution for housing the rapidly increasing number of older people all over the world [2]. Vasunilashorn et al. (2012) pointed out that the studies of aging in place covered a variety of areas, such as housing, community, social service and health [3]. Urban environment has been identified as an important element that supports older people’s ability to actively age and age in place [1,4]. The specific neighborhood environment has been found as an important determinant that affects active aging among older people [5].

Community facilities are an important element of an age-friendly city [6], as they have a close relationship with older people’s daily life. The provision and easy access to different types of community facilities could help improve older people’s physical health status [7] and enhance their well-being and social benefits [8]. The lack of community amenities has been identified as the main neighborhood design characteristic that affects older people’s ability to actively age [9,10] and age in place [11]. Previous studies also pointed out that community facilities are important variables that may be associated with older people’s neighborhood satisfaction and life satisfaction [12,13]. However, there is inadequate knowledge for urban planners to provide suitable community facilities to achieve age-friendly and healthy communities [14].

Previous studies have tried to explore the relationship between the planning and individuals’ usage patterns of community facilities. Some studies focused on the design quality of community facilities, such as the size and diversity [15,16]. Existing studies also examined the accessibility to community facilities, such as their proximity to home [9] or the walkability and street network surrounding them [13,17]. In addition, most of the studies focused on a single type of community facility, such as parks [18], recreational facilities [14] or welfare facilities [19]. Few studies have tried to provide a systematic framework to examine older people’s usage pattern of different types of community facilities.

Older people’s subjective well-being is important to achieve healthy aging [20]. Previous studies have found that the built environment of a community could affect older people’s well-being, such as residential density [21], housing condition [22], outdoor spaces [23] and street connectivity [24]. Community facility has been identified as an essential component that may contribute to people’s well-being [25]. However, these studies mainly focused on the provision of community facilities or access to community facilities [26,27,28]. How older people’s usage patterns and satisfaction with community facilities affect their well-being is still unclear. This study aims to address this research gap.

Moreover, Hong Kong is a high-density city with a unique urban form and culture. Thus, the findings of studies conducted in Western countries cannot be directly applied to eastern countries, such as Hong Kong [25]. Vine et al. (2012) found that the reliance on motor vehicles was an important issue that affected older people’s access to local amenities in Australia [29]. However, a lower proportion of older people in Hong Kong had private cars [30]; thus, it may not be the main issue that affects their usage of community facilities. Furthermore, a study in European cities pointed out that green spaces in care facilities were important to older people. Due to limited land resources, community facilities in Hong Kong usually do not have their own green spaces. Thus, it is important to explore older people’s usage and satisfaction level with community facilities in the specific context of a high-density Asian city, such as Hong Kong.

The proportion of people aged 65+ in Hong Kong was approximately 18% in 2019 and is projected to increase to 35% in 2069 [31]. A large number of Hong Kong older adults live in urban old districts due to financial constraints or the need to maintain their social network [32]. These districts usually have an insufficient provision of community facilities. To improve the built environment of neighborhoods in the urban old districts, the Urban Renewal Authority has carried out urban renewal projects since its establishment in 2001. This provides a good opportunity to redistribute community facility resources and increase older people’s user satisfaction. Therefore, it is necessary to further explore the relationship between the planning and design of community facilities and older people’s usage and satisfaction in Hong Kong.

Given the above, the objectives of this research study were: (1) to identify the factors that influence older people’s usage pattern of different types of community facilities; (2) to examine the association between the usage of a community facility, planning and design of a community facility and older people’s satisfaction level with using different types of community facilities; (3) to explore the relationship between the provision of community facilities, older people’s usage pattern and well-being.

## 2. Conceptual Framework

### 2.1. Older People’s Need for Community Facilities and Well-Being

Community facilities have a close relationship with people’s daily life, as they provide spaces for their everyday activities [33]. Older people usually rely on public community facilities for recreational and social interaction purposes [34]. Having various community facilities in the local neighborhood reduces older people’s need to visit other neighborhoods for everyday living and decreases their reliance on motor vehicles [29]. The length of stay in recreational facilities has been found to be significantly correlated with mental health and social functioning [35]. Thus, Scharlach and Lehning (2013) pointed out that facilities supporting older people’s daily activities are one the fundamental elements of age-friendly communities [36]. The presence of facilities tailored to older people is also one of the essential neighborhood characteristics to support aging in place [37].

The neighborhood and its built environment are important for people’s well-being [38]. Well-being has been associated with a variety of neighborhood characteristics, such as access to services [39], open space and greenery [40], safety and noise [41] and quality of facilities [42]. Community facilities are one of the key components of neighborhoods that may impact on residents’ well-being. It has been found that providing new services and amenities in urban renewal areas could help promote residents’ well-being [43]. Musa et al. (2018) also pointed out that the quality of services and local facilities had a significant impact on people’s social well-being [44]. Similar findings were also reported by Almedom (2005) who concluded that access to public goods and services and amenities could contribute to people’s social capital and mental health [45]. In Canada, access to facilities was considered an important contributor to the quality of life and well-being by local residents [46]. The importance of community facilities has been recognized by various scholars, thus leading to them being included in questionnaires assessing community well-being [47].

### 2.2. Factors Influencing Usage of and Satisfaction with Community Facilities

Previous studies have proved that the provision of community facilities is a fundamental requirement for community planning, and it is necessary to provide adequate community facilities for residents. A study conducted in the UK found that a higher number of recreational facilities could encourage people’s usage and promote physical activity level [48]. Tang (2017) also found that adequate provision of community facilities and services could help with the livability and sustainability of the neighborhood in a high-density city [49]. In contrast, Koohsari et al. (2013) reported that too many community facilities would be associated with less walking [50]. The effect of the provision of community facilities on older people’s usage and satisfaction level still remains to be investigated.

Size was also an important variable that affects older people’s use of community facilities. Larger community facilities usually have enough space for users to conduct different activities at the same time [51]. Users’ satisfaction level could also be increased by providing a comfortable and age-friendly activity space [52]. The larger size of community facilities could also encourage people to engage in more physical activity [53].

The diversity of community facilities has also been identified as an essential element to neighborhood planning. It has been found that the diversity of community facilities could improve the walkability of the neighborhood [54]. Different types of community facilities have their own functions and meet older people’s daily life needs, which should be considered during the planning process [55]. They can also meet the requirements of diverse population groups and different communities.

Several studies also highlighted that community facilities should be in proximity of users’ homes [56,57]. It is suggested that community facilities, such as commercial or recreational facilities, should be located within an acceptable walking distance, so that people can easily access and participate in various activities that promote health [58].

The proximity to public transport is also an important consideration in the planning of community facilities. Previous studies have proved that access to public transport has a significant impact on older people [9,59] because it provides them the opportunity to reach their preferred places far away and promotes their physical activity level [60]. Clarke and Nieuwenhuijsen (2009) claimed that poor access to public transport was one of the environmental barriers for older people to age in place and maintain their health [61].

There is also evidence that community facilities should be in proximity of other amenities, which could increase the opportunity for older people to use other amenities [62]. In Hong Kong, more amenities around leisure facilities were found to promote older people’s usage of leisure facilities [63]. Thus, it is important for planners to consider the planning of different types of community facilities in a comprehensive way.

Apart from examining the planning and design quality of community facilities, to understand their usage pattern, older people’s purpose of using community facilities should also be considered. In fact, older people’s purpose for visiting specific community facilities was one of the critical variables that determined whether they would participate in leisure activities. In addition, community facilities that could fulfill people’s autonomy needs were more likely to be visited multiple times [64].

**Figure 1:**
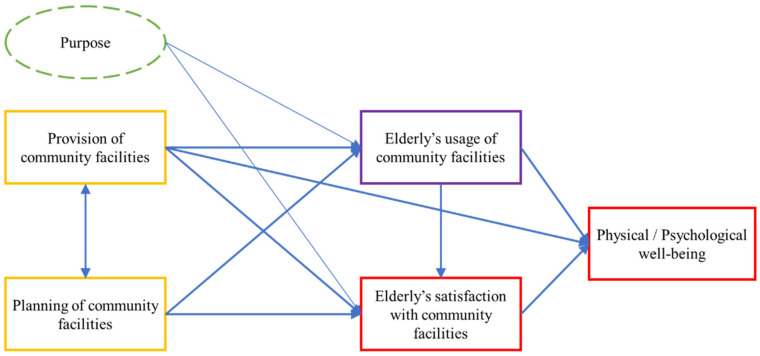
Conceptual framework of the relationship between planning, usage and satisfaction with community facilities.

Based on an analysis of the extant literature, this study proposes a conceptual framework (Figure 1) demonstrating the relationships among the provision and planning of community facilities, older people’s usage and satisfaction level. It is hypothesized that the provision of community facilities and planning and design quality of community facilities, as well as purpose, would impact on both older people’s usage and satisfaction level with the community facilities. In addition, older people’s usage of community facilities could also affect their satisfaction level. Finally, the provision of community facilities, older people’s usage and satisfaction level with community facilities could be associated with their physical and psychological well-being.

## 3. Research Methods

### 3.1. Site Selection

This study selected the Kwun Tong and Sham Shui Po districts based on the following reasons: (1) in 2016, the two districts had a high number and proportion of older people, indicating a stronger need for community facilities tailored to older people; (2) the two districts are both districts with low socio-economic status; thus, older people living in these districts rely more on public community facilities; (3) the two districts are old districts with undergoing urban renewal projects conducted by the Urban Renewal Authority, which provides an opportunity to improve the provision and planning quality of community facilities. The selected districts can represent the typical usage pattern and need for community facilities by older people living in urban old districts. The study districts do not include the Kwun Tong industrial area due to very few older people living there. The details of profiles of the Kwun Tong and Sham Shui Po districts are presented in Table 1.

### 3.2. Spatial Analysis

**Figure 2:**
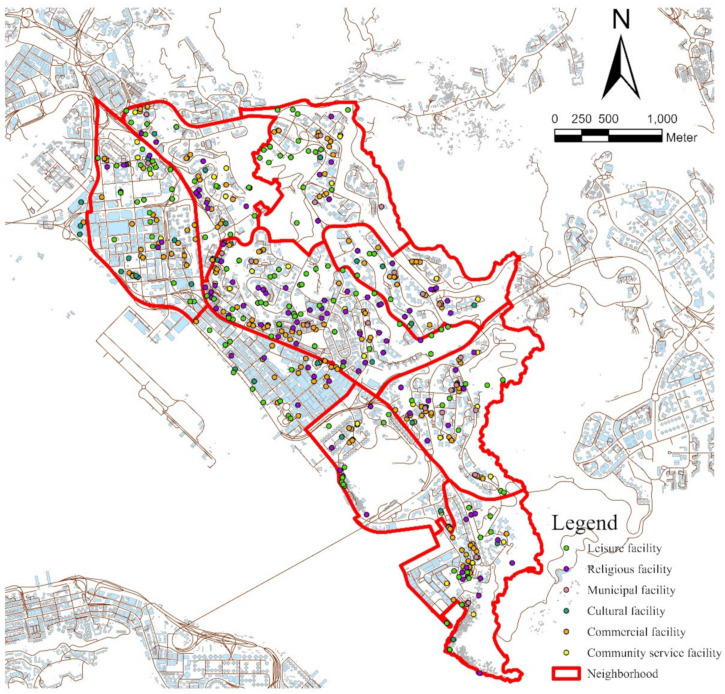
Spatial distribution of community facilities in Kwun Tong district.

**Figure 3:**
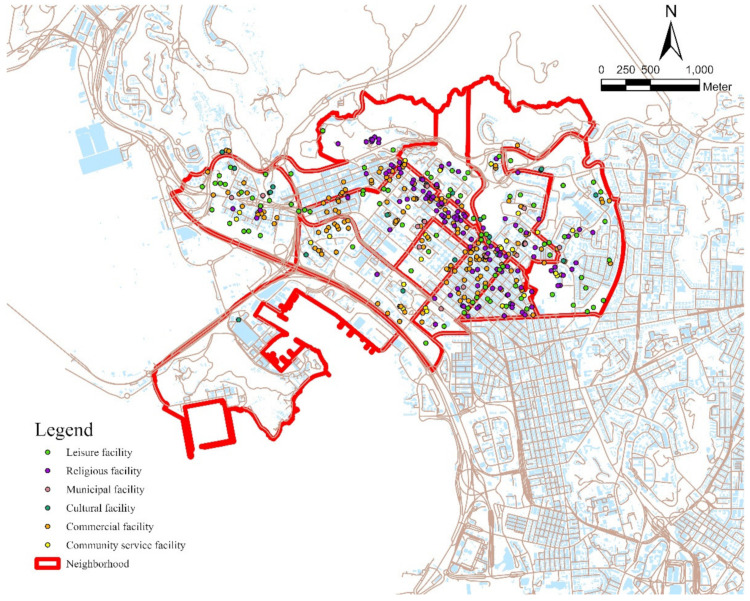
Spatial distribution of community facilities in Sham Shui Po district.

The spatial analysis aimed to characterize the spatial distribution of community facilities in the neighborhood. Two variables were calculated using this method: the number of different types of community facilities in the neighborhood and the ratio of older residents per community facility in the neighborhood, which is defined as the number of older people per community facility [32]. This unit matches the Hong Kong Planning Standards and Guidelines, and a higher ratio of residents to facility usually indicates insufficient provision. Tertiary Planning Units (TPUs) are used to define neighborhoods. TPUs are the smallest planning unit and have been used in previous studies [65,66,67]. Digital maps and a community facility database were obtained from the Survey and Mapping Office of Lands Department as of August 2020. In addition, the 2016 Tertiary Planning Unit (TPU) boundary data were obtained from the Planning Department. Table 2 describes the six different types of community facilities used in this study. The spatial distributions of community facilities in the Kwun Tong and Sham Shui Po districts are presented in Figure 2 and Figure 3, respectively.

### 3.3. Questionnaire Survey

The subjects of the questionnaire survey were older people aged over 55 who were willing to participate in the survey in the two study districts. The age eligibility criterion was determined based on the fact that many Hong Kong centers for older people provide services to people aged 55 years and over. The survey was conducted from April 2021 to January 2022. The recruitment of participants was conducted in the places that older people may frequently use, such as parks, podium gardens of housing estates and elderly centers. Older people were randomly invited to participate in the survey.

The questionnaire consisted of three parts (File S1). The first part collected information on older people’s usage patterns of six different types of community facilities. It included older people’s frequency of visitation (response options: 1–2 times per week; 3–5 per week; 5–7 per week; >7 times per week) and length of stay (response options: <15 min; 15–30 min; 30–60 min; >60 min) in specific types of community facilities. In addition, information on older people’s purpose of visiting different types of community facilities was also collected. The second part focused on older people’s perception and preference of planning and design features of community facilities. Older people were asked to rate each planning and design factor on a five-point Likert scale (1 = strongly disagree … 3 = neutral … 5 = strongly agree). The third section consisted of older people’s evaluation of their satisfaction level with using specific community facilities on a five-point Likert-like scale (1 = very unsatisfactory … 3 = neutral … 5 = very satisfactory). The measurements of older people’s physical well-being and psychological well-being were mainly based on those used in a previous study [68], which was also conducted in Hong Kong and whose validity in assessing older adults’ well-being was proven. The details of the measurement of physical and psychological well-being are presented in Appendix A. In addition, older people’s basic socio-demographic characteristics, such as age and gender, were also collected during the questionnaire survey.

### 3.4. Data Analysis

First, this study used the descriptive analysis method to understand older people’s usage pattern and preference of community facilities, such as frequency, length of stay, purpose of visiting and satisfaction level of different types of community facilities. Second, five models were performed to estimate the relationship among community planning, usage and older people’s well-being. Generalized estimating equation (GEE) models were used to estimate the associations between the planning and design of community facilities and older people’s usage pattern and well-being. This method was suitable for this study because it could help avoid the specific effects of the planning and design of the community facility of each neighborhood (TPU) [69]. 

The first and second models aimed to identify the influential factors that affect older people’s usage pattern of community facilities. The dependent variables were the frequency of visitation of a specific type of community facility and the length of stay in the community facility, respectively, which were ordinal variables. The independent variables included the provision of community facilities (number and ratio of residents to a facility), the planning and design quality and the purpose of visiting a community facility.

The third model aimed to explore the influential factors that affect older people’s satisfaction level with using the community facility. The dependent variables were older people’s satisfaction level with using a specific type of community facility, which was also an ordinal variable. The independent variables included the usage pattern of a community facility (frequency and length of stay), the provision of community facilities (number and ratio of residents to a facility), the planning and design quality and the purpose of visiting a community facility.

The fourth and fifth models intended to illustrate the relationship between the community facility and older people’s well-being. The two dependent variables were physical well-being and psychological well-being, respectively, which were continuous variables. The independent variables included the usage pattern of a community facility (frequency, length of stay and satisfaction level) and the provision of community facilities (number and ratio of residents to a facility).

The socio-demographic characteristics (age and gender) and districts were included in the models as covariates. The different analysis models (linear model and logit model) were selected based on the types of dependent variables. All statistical analyses were conducted using SPSS Statistics 25 software (IBM Inc., Armonk, NY, USA).

## 4. Results

### 4.1. Characteristics of Respondents and Usage Pattern of Community Facilities

In this study, around 2000 older people living in study districts were invited to participate in the questionnaire survey. A total of 509 of them returned the filled questionnaires, and 12 participants were excluded due to incomplete questionnaires. Finally, a total number of 497 questionnaires were included in the analysis: 261 from the Kwun Tong district and 236 from the Sham Shui Po district. Table 3 shows the profiles of the respondents and their usage pattern of community facilities. Most of the respondents were aged 61 to 80 years old. Commercial, municipal and leisure facilities were the three most commonly used types of community facilities, with more than 400 users among 497 respondents. This study also indicated that more than 70% of respondents chose to visit all six types of community facilities by walking, and more than 60% of respondents found it acceptable to walk up to 20 min to a facility.

This study indicated that leisure and municipal facilities were the ones most frequently visited by older people, with 53.7% and 39.9% of respondents visiting them more than 6 times a week, respectively, followed by 27.4% of respondents visiting commercial facilities. Religious facilities were one of the least frequently visited locations by older people, with 73.0% of respondents visiting them only 1–2 times per week. In terms of length of stay, older people usually spent more time at religious and leisure facilities per visit. We found that 58.4% and 45.0% of respondents stayed for more than one hour at religious facilities and leisure facilities, respectively. In addition, we found that older people were most satisfied with using religious and commercial facilities. The percentages of users who were satisfied or very satisfied were 77.4% and 71.7% for religious facilities and commercial facilities, respectively, followed by 69.0% for leisure facilities.

The purposes of using community facilities varied among the different types of facilities. A total of 88.3% and 94.2% of respondents visited commercial and municipal facilities for basic life needs, indicating that these facilities had a single function. With regard to community service and leisure facilities, although more than half of the users visited these facilities for social interaction (50.7%) or physical exercises (68.3%), there were still some other purposes that cannot be ignored. In addition, the purposes of visiting cultural and religious facilities were quite diverse, showing that these two types of facilities were multi-functional. Furthermore, the findings suggested that older people may visit more frequently the community facilities that are related to their daily life. Leisure, municipal and commercial facilities were the three most frequently visited facilities, which most older people visited for basic life needs and physical exercises.

### 4.2. Influential Factors on the Older People’s Usage Pattern of Community Facilities

Table 4 and Table 5 present the results of generalized estimating equation (GEE) models for the frequency of visitation of specific types of community facilities and length of stay in community facilities, respectively. The study found that the number of commercial facilities in a neighborhood was negatively related to older people’s frequency of visiting commercial facilities (OR = 0.963, *p* < 0.001) and length of stay in a commercial facility (OR = 0.947, *p* < 0.001). The study also indicated that the ratio of residents to a commercial facility was negatively correlated to older people’s frequency of visiting commercial facilities (OR = 0.325, *p* < 0.001) and length of stay in a commercial facility (OR = 0.459, *p* < 0.05). Proximity to other amenities (OR = 1.255, *p* < 0.05) was positively associated with older people’s s frequency of visiting commercial facilities. In addition, an adequate number of commercial facilities in the neighborhood (OR = 1.554, *p* < 0.01) and visiting commercial facilities for entertainment purposes (OR = 2.085, *p* < 0.05) were positively associated with older people’s length of stay in commercial facilities.

For community service facilities, an adequate number of community service facilities in the neighborhood (OR = 1.388, *p* < 0.05), perceived proximity to home (OR = 1.432, *p* < 0.05) and proximity to other amenities (OR = 1.436, *p* < 0.05) were positively related to the frequency of visitation, which means that if there were enough community service facilities in the neighborhood, or a community service facility was close to home or other amenities, they may visit a community service facility more frequently. In addition, using a community service facility for the purposes of accompanying family and friends (OR = 4.116, *p* < 0.05) and volunteer work (OR = 1.810, *p* < 0.05) was positively associated with older people’s frequency of visiting community service facilities. Furthermore, this study found that the perceived proximity to public transport (OR = 1.824, *p* < 0.01) was positively associated with older people’s length of stay in community service facilities. Using the facility for basic life needs (OR = 0.355, *p* < 0.01) was negatively associated with older people’s length of stay in a community service facility.

Older people’s frequency of visiting cultural facilities was only significantly associated with the purpose of accompanying family and friends (OR = 4.124, *p* < 0.05). In addition, the length of stay in cultural facilities had a positive relationship with the perceived diversity of facilities (OR = 1.860, *p* < 0.001) and the purpose of entertainment (OR = 2.221, *p* < 0.05). It was also indicated that the ratio of residents to a cultural facility (OR = 0.928, *p* < 0.01) had a negative relationship with the length of stay in cultural facilities.

With regard to municipal facilities, an adequate number of community service facilities in the neighborhood (OR = 1.257, *p* < 0.05) and visiting for the purpose of basic life needs (OR = 3.081, *p* < 0.001) were significantly associated with older people’s frequency of visiting municipal facilities. In addition, the number of municipal facilities in the neighborhood (OR = 0.824, *p* < 0.05) had a negative relationship with older people’s frequency of visiting municipal facilities. The purpose of accompanying family and friends (OR = 2.309, *p* < 0.001) was also found to be positively related to older people’s length of stay in municipal facilities, while the number of municipal facilities in the neighborhood was negatively associated with the length of stay (OR = 0.916, *p* < 0.01).

We found that only the purpose of accompanying family and friends (OR = 1.618, *p* < 0.05) was positively associated with older people’s visitation frequency of leisure facilities. In terms of the length of stay in leisure facilities, facility size (OR = 1.509, *p* < 0.001) and visiting the facility for the purpose of physical exercise (OR = 2.565, *p* < 0.001), accompanying family and friends (OR = 1.540, *p* < 0.05) and entertainment (OR = 1.829, *p* < 0.001) had positive relationships with older people’s length of stay, while of the number of leisure facilities in the neighborhood (OR = 0.983, *p* < 0.001), the ratio of residents to a leisure facility (OR = 0.770, *p* < 0.001) and the perceived diversity of facilities (OR = 0.683, *p* < 0.05) negatively contributed to older people’s length of stay in leisure facilities.

For religious facilities, older people’s purpose of visiting the facility for accompanying family and friends (OR = 5.688, *p* < 0.01) or volunteer work (OR = 6.082, *p* < 0.001) had positive associations with older people’s frequency of visiting religious facilities. Furthermore, the purpose of social interaction (OR = 3.184, *p* < 0.05) was found to be positively associated with older people’s length of stay in religious facilities. The perceived diversity of facilities (OR = 0.481, *p* < 0.01) was also found to be negatively correlated with older people’s length of stay in religious facilities.

### 4.3. Older People’s Usage Pattern and Satisfaction with Using Community Facilities

Table 6 presents the results of generalized estimating equation (GEE) models for older people’s satisfaction level with using community facilities. Older people’s satisfaction level with using commercial facilities was positively associated with the number of commercial facilities in the neighborhood (OR = 1.020, *p* < 0.01), the ratio of residents to a commercial facility (OR = 2.097, *p* < 0.01), diversity (OR = 2.126, *p* < 0.001) and proximity to other amenities (OR = 1.740, *p* < 0.001).

This study also found that the ratio of residents to a commercial facility (OR = 1.555, *p* < 0.01) had positive relationships with satisfaction with using community service facilities. The purpose of visiting the facility for accompanying family and friends (OR = 0.391, *p* < 0.01) was found to be negatively correlated with satisfaction with using community service facilities.

For cultural facilities, proximity to other amenities (OR = 3.371, *p* < 0.001) was positively related to satisfaction with using cultural facilities. In addition, the results indicated that the facility size (OR = 1.758, *p* < 0.01), perceived diversity of facilities (OR = 1.957, *p* < 0.001) and the purpose of visiting the facility for basic life needs (OR = 2.062, *p* < 0.001) had positive relationship with older people’s satisfaction with using municipal facilities.

This study indicated that older people’s satisfaction level with using leisure facilities was positively associated with the facility size (OR = 1.840, *p* < 0.01) and perceived diversity of facilities (OR = 1.632, *p* < 0.01) and perceived proximity to home (OR = 1.289, *p* < 0.01). The number of leisure facilities in the neighborhood (OR = 0.983, *p* < 0.05) had a negative relationship with older people’s satisfaction level with using leisure facilities.

Furthermore, this study found that proximity to public transport (OR = 4.627, *p* < 0.05) and the purpose of acquiring new knowledge (OR = 2.736, *p* < 0.05) were positively associated with satisfaction with using religious facilities, while the adequate number (OR = 0.468, *p* < 0.05) and purpose of social interaction (OR = 0.268, *p* < 0.05) showed negative associations.

### 4.4. Relationship between Older People’s Usage Pattern, Satisfaction with Community Facilities and Well-Being

Table 7 reports the results of the generalized estimating equation (GEE) models of older people’s physical well-being. Older people’s frequency of visiting commercial facilities (Coefficient = 0.143, *p* < 0.01), community service facilities (Coefficient = 0.131, *p* < 0.05), municipal facilities (Coefficient = 0.137, *p* < 0.01) and religious facilities (Coefficient = 0.250, *p* < 0.001) and the length of stay in a municipal facility (Coefficient = 0.098, *p* < 0.05) were positively related with physical well-being. In addition, the satisfaction level with using commercial facilities (Coefficient = 0.139, *p* < 0.05) and municipal facilities (Coefficient = 0.146, *p* < 0.05) also positively contributed to older people’s physical well-being. In terms of the provision of community facilities, the number of cultural facilities (Coefficient = −0.068, *p* < 0.05) and religious facilities (Coefficient = −0.021, *p* < 0.001) and the ratio of residents to a religious facility (Coefficient = −0.059, *p* < 0.01) were negatively associated with older people’s physical well-being.

Table 8 reports the results of the generalized estimating equation (GEE) models of older people’s psychological well-being. Older people’s frequency of visiting municipal facilities (Coefficient = 0.145, *p* < 0.05) and the length of stay in a commercial facility (Coefficient = 0.107, *p* < 0.05), cultural facility (Coefficient = 0.150, *p* < 0.01) and religious facility (Coefficient = 0.339, *p* < 0.001) were positively associated with their psychological well-being. Except for religious facilities, the results indicated that higher satisfaction level with using community facilities could help improve the elderly’s psychological well-being. In addition, the number of community service facilities (Coefficient = −0.058, *p* < 0.05) was negatively related to older people’s psychological well-being.

## 5. Discussion

### 5.1. Older People’s Usage Pattern of Community Facilities, Purpose of Visitation and Daily Life

This study suggests that older people’s usage of community facilities and frequency of visiting community facilities mainly depend on the functions of community facilities or the purpose of using community facilities. Community facilities are closely related to older people’s daily life, in the sense that they provide different types of support that enhances older people’s ability to age in place [6]. The results from this study show that commercial, municipal and leisure facilities are the most commonly used facilities. This may be due to these facilities having the purpose of satisfying the basic life needs (buying food and vegetables) and providing opportunities for physical exercise. Physiological and safety needs are two basic needs, which are closely related to people’s daily life [70]. Compared to other community facilities, which mainly provide opportunities for social activities, these three types of facilities are commonly used by the majority of older people. 

In addition, older people’s purpose of visiting community facilities was identified as an important factor that affects older people’s frequency of visiting community facilities. The main reason that makes older people visit community facilities is the actual type of purpose of visiting the facilities or the activities that the community facilities provide. In contrast, the provision and quality of the community facilities only have an effect on visitation frequency of some specific types of community facilities. This study also shows that older people may visit community service, cultural, leisure and religious facilities more frequently if their main motivation is engagement in social activities, such as accompanying family and friends or volunteer work. Older people seem to rely on these facilities to participate in social activities to maintain their social network and recognize their self-worth [71].

We hypothesized that older people’s length of stay in community facilities would be affected by the planning and design quality of the facilities, as well as the purpose of using the facilities. This study shows that, although the purpose of visiting and using community facilities is important to older people’s visitation frequency of community facilities, it does not affect the length of stay to the same extent. The facility’s quality attributes, such as size, adequate number in the neighborhood, diversity or location, may have a significant impact on older people’s length of stay in specific community facilities, which emphasizes the importance of providing high-quality community facilities. An interesting finding from this study is that a larger number of commercial and municipal facilities in the neighborhood would decrease older people’s length of stay in the facility. This may be because more commercial and municipal facilities in the neighborhood provide more alternative choices for older people to engage in shopping and other activities, which may, in turn, decrease their length of stay in particular facilities [50].

### 5.2. Planning and Design Quality of Community Facilities and Satisfaction Level

This study suggests that older people’s satisfaction with using community facilities is mainly affected by the planning and design quality of community facilities. Older people’s purpose of visiting community facilities plays a less important role. Although older people’s purpose of visiting community facilities may affect the frequency of using the facilities and the length of stay, this does not mean that they will feel satisfied with using the facilities. Ho et al. (2021) pointed out that the satisfaction level with community facilities is mainly the result of the quality of the facility itself rather than other factors [72]. Thus, to enhance older people’s satisfaction level, the most important strategy is to improve the quality of community facilities. In addition, the findings show that the usage pattern of community facilities and the provision of community facilities may have little effect on older people’s satisfaction with specific facilities, as there is no significant association between older people’s usage pattern and satisfaction with community facilities. Neal et al. (2007) pointed out that the length of stay may not have a direct effect on people’s satisfaction level with services but may instead play a moderating role [73]. Thus, it is still important to take special care in satisfying the needs of those who visit community facilities frequently or stay for a longer time. Zhang et al. (2017) also claimed that the quality of community facilities is more important to people’s satisfaction and well-being than their quantity [74]. Thus, only providing enough community facilities, which is usually the main objective of existing planning standards and guidelines, is insufficient to make older people feel satisfied with the community facilities.

The planning and design quality of community facilities is the most critical factor that contributes to older people’s satisfaction level with using community facilities. Size plays an important role in the satisfaction with using municipal and leisure facilities. Larger community facilities provide more space for different activities [51]. Due to the limited land resources in Hong Kong, the facilities may not provide enough space to conduct different types of activities for older users. This is especially relevant for a leisure facility, which is multi-functional. Larger community facilities could increase older people’s comfort levels and make them feel more satisfied with using community facilities [52]. The diversity of community facilities could also increase older people’s satisfaction level, especially in relation to commercial, municipal and leisure facilities. A higher level of diversity in community facilities means that older people could access most facilities that meet their needs in their own neighborhood. This would create more opportunities for older residents to walk for utilitarian purposes [9].

The location of community facilities is also an essential factor for older people’s satisfaction level. This study suggests that if commercial and cultural facilities are close to other amenities, older people may feel more satisfied. The result is in line with a previous study, which found that older people living in high-density cities prefer to link different types of activities when they go outside [63]. This may be due to older people being accustomed to attending various daily activities in one trip, such as having breakfast, buying food and vegetables, visiting an elderly community center and library [57]. Thus, proximity to other amenities could facilitate the visitation of other facilities. In fact, previous studies have found positive associations between the presence of other amenities, walking and physical activity [75]. Proximity to public transport could also benefit older people’s satisfaction level with using religious facilities. This study suggests that, for religious facilities, older people may be willing to visit the facilities far away from home. Thus, public transport is essential for older people to visit these community facilities. Temelová and Dvořáková (2012) indicate that older people often use public transport to participate in social activities, such as visiting relatives and friends [12]. In this study, social activities were the main reason for older people to visit religious facilities. Thus, proximity to public transport could increase older people’s satisfaction level with using religious facilities.

### 5.3. Older People’s Use of Community Facility and Well-Being

This study also explored the relationships among the provision of community facilities, older people’s usage pattern of community facilities and their physical and psychological well-being. The results indicate that higher visitation frequency and satisfaction level with using commercial and municipal facilities may help promote older people’s physical well-being. Commercial and municipal facilities are the two types of community facilities that are most commonly used by older people, as they are directly related to their daily life. Most older people visit these facilities to buy food and vegetables or for other daily necessities, which are essential for aging in place [76]. Thus, frequently visiting commercial and municipal facilities could help increase older people’s walking, which may benefit their physical well-being. 

In addition, higher frequencies of visiting municipal facilities and longer stays in commercial, cultural and religious facilities were found to be positively associated with older people’s psychological well-being. This may be due to social interaction being the main reason for older people to visit these facilities, which is known to positively affect people’s psychological well-being, especially among older people [77]. In addition, older people also participate in volunteer work at, or accompany family or friends to, religious facilities. This could help them maintain their relationship with family and friends and enhance their self-worth, which could also promote their psychological well-being [78,79]. With respect to municipal facilities, older people in Hong Kong prefer to visit markets near their home and spend time talking with stallholders or other older people. This could also help them maintain their social network in the neighborhood, which has been linked to better psychological well-being [79].

This study found that the provision of community facilities may not have as obvious an effect as usage patterns on older people’s well-being, especially psychological well-being. An interesting finding is that older people living in neighborhoods with a smaller number of community facilities may have higher levels of physical and psychological well-being. A possible reason is that older people’s need for community facilities is mainly based on their daily life needs, which are not reduced by lower provision. Figure 2 and Figure 3 illustrate that the distributions of community facilities are not even among the different neighborhoods. Thus, older adults with fewer community facilities in their neighborhood may visit facilities far away from home [56]. This may increase older people’s walking, which may also promote their well-being [80]. In general, only focusing on the provision of community facilities is not sufficient to promote older people’s physical and psychological well-being.

### 5.4. Urban Planning Policy Recommendations

This study identified the key factors that affect older people’s usage pattern and satisfaction level with using community facilities. As such, it provides valuable insights for urban planners and policy makers that can be incorporated into the planning of community facilities to encourage older people’s usage of community facilities and increase their satisfaction. In general, this study indicates that the existing planning policies, which mainly focus on the quantity of the community facilities, are insufficient to increase older people’s satisfaction level and improve their well-being. It is important for planners to pay attention to older people’s purposes of using community facilities and the quality of community facilities. Older people visit different types of community facilities for different purposes. These purposes are the main driving factors that affect the visitation frequency of community facilities. Thus, it is important for planners to provide a variety of types of community facilities in the neighborhood to meet older people’s different needs, so that they can satisfy their daily needs in their own neighborhood. In addition, planners should pay attention to the number of commercial and municipal facilities in the neighborhood to avoid disordered competition and lack of space for other community facilities.

With regard to commercial facilities, it is important to ensure an adequate number of facilities to meet older people’s shopping needs. The diversity of commercial facilities is also important for older people. Community planning should try to provide space for diverse types of commercial facilities, such as convenience stores, supermarkets or shopping malls. The location of commercial facilities should be near other community amenities, so that older people can link various destinations in a single trip. In terms of community service facilities, the key planning strategy is to ensure the facility is in proximity of housing estates, public transport stations and other community amenities. Public transport services could help older people visit facilities outside of their neighborhood for social interaction activities. Furthermore, the adequate number of community service facilities is also important. Older people use community service facilities to conduct a variety of activities; therefore, planners should try to ensure the adequate number of community service facilities.

The planning of cultural facilities should focus on the diversity and proximity to other amenities. It is important to provide different types of cultural facilities in the neighborhood, such as public libraries, museums or cultural centers, where older people can enjoy Cantonese Opera. The location of these cultural facilities should be in proximity to other community amenities. This could help older people participate in other activities, such as shopping in commercial facilities or relaxing in leisure facilities after visiting cultural facilities. With regard to municipal facilities, the size and diversity are important contributors to older people’s satisfaction. As municipal facilities are the place that older people usually visit to buy fresh produce, it is essential to provide enough space to allow more merchants to provide service in the facility. Larger wet markets (a type of municipal facility) could also make older people feel more comfortable. A higher level of diversity in municipal facilities could provide more alternative choices for older people, which may increase their satisfaction with shopping in this type of facility.

Our study suggests that older people need a variety of larger leisure facilities in proximity to their home. Larger leisure facilities usually provide enough space for older people to participate in group activities, such as Tai Chi or dancing. They may also provide opportunities for older people to enjoy more fitness facilities. In addition, different types of leisure facilities could provide different services or activities for older people and alternative choices. Planners should try to provide different types of leisure facilities, such as parks, playgrounds or sitting-out areas in the local neighborhood. Interestingly, this study suggests that leisure facilities are the only type of facility that older people require to be located near their home; thus, the planning of leisure facilities should consider the distance of such facilities from the housing estates. As for religious facilities, the proximity to public transport was significantly related to older people’s satisfaction with such facilities. Therefore, planners should try and locate religious facilities close to public transport stations, so that older people could easily access them by public transport. Providing an adequate number of religious facilities is also important.

### 5.5. Study Limitations

Several limitations of this study should be pointed out. First, this study is a cross-sectional study; thus, the causal effects cannot be inferred. Second, due to the limited human resources, only a limited sample size of older people is included in the study, which may not be representative of the target population. Third, older people’s self-reports of the usage pattern of community facilities are collected from a questionnaire survey, which is subject to recall bias.

## 6. Conclusions

This study builds a conceptual framework to better understand the factors that affect older people’s usage and satisfaction level with using six different types of community facilities. It also explores the relationship between the provision and usage patterns of community facilities and older people’s physical and psychological well-being.

This study found that older people’s usage and frequency of visiting community facilities mainly depended on the purpose of the visits and the activities the facilities provided rather than the planning and design quality variables. In addition, the factors explaining older people’s length of stay varied across the different types of community facilities. This study also suggested that older people’s satisfaction level with using community facilities was mainly associated with the planning and design quality of the facilities rather than the purpose of visitation or usage pattern. Furthermore, this study highlighted that older people’s physical and psychological well-being was more clearly and more strongly related to their satisfaction level with using different types of community facilities.

This study makes contributions to both the theory and practice of planning and design of community facilities to achieve age-friendly communities. The novelty of this study is in developing a model, which examines the relationship among the provision, planning and design and usage of community facilities and older people’s well-being within the context of dense, older urban neighborhoods. It fulfills the knowledge gap of how planning and design of different types of community facilities would affect older people’s usage and well-being. The findings of this study can contribute to expanding the urban planners’ knowledge of the factors related to older residents’ satisfaction with community facilities and well-being and their needs associated with different types of community facilities. From a practical perspective, the existing planning standards and guidelines mainly set the provision ratio of community facilities; the findings of this study could provide supplementary reference for the planning and design principles of different types of community facilities. The empirical results of this study could provide guidelines for urban planners or policy makers in the planning and distribution of community facilities to promote older people’s well-being and achieve age-friendly communities, especially in an Asian area with a similar urban context, such as Shanghai or Singapore.

## Figures and Tables

**Table 1:** Profiles of two selected study districts.

	Kwun Tong District	Sham Shui Po District
Population of older people	111 259	64 473
Proportion of older people	17.2% (1st among 18 districts)	15.9% (8th among 18 districts)
Median Monthly Domestic Household Income	20,160 (17th among 18 districts)	20,000 (18th among 18 districts)
Poverty rate (pre-intervention)	28.8% (1st among 18 districts)	26.5% (5th among 18 districts)

**Table 2:** Community facility type and description.

Facility Type	Sham Shui Po District
Commercial facility	Convenient store; mall/shopping center/commercial complex; supermarket
Community service facility	Community center/community hall/elderly center/welfare center/family service center
Cultural facility	City hall/Town hall/civic center/exhibition venue; library; cinema
Municipal facility	Municipal complex; market
Leisure facility	Park; playground; minor open space (Passive)
Religious facility	Church; monastery/nunnery; mosque; other religious places; seminary; synagogue; temple

**Table 3:** Profile of the respondents and usage pattern of community facilities.

	Commercial Facility (*n* = 444)	Community Service Facility (*n* = 288)	Cultural Facility (*n* = 149)	Municipal Facility (*n* = 413)	Leisure Facility (*n* = 438)	Religious Facility (*n* = 137)
**Age**						
55–60	31	13	12	27	28	4
	7.00%	4.50%	8.10%	6.50%	6.40%	2.90%
61–70	165	90	59	151	158	35
	37.20%	31.30%	39.60%	36.60%	36.10%	25.50%
71–80	156	117	59	152	158	55
	35.10%	40.60%	39.60%	36.80%	36.10%	40.10%
81–90	87	63	18	79	88	39
	19.60%	21.90%	12.10%	19.10%	20.10%	28.50%
>90	5	5	1	4	6	4
	1.10%	1.70%	0.70%	1.00%	1.40%	2.90%
**Gender**						
Male	179	108	67	167	185	47
	40.30%	37.50%	45.00%	40.40%	42.20%	34.30%
Female	265	180	82	246	253	90
	59.70%	62.50%	55.00%	59.60%	57.80%	65.70%
**Frequency**						
1–2 times	129	133	84	98	63	100
	29.10%	46.20%	56.40%	23.70%	14.40%	73.00%
3–5 times	193	98	39	151	140	27
	43.50%	34.00%	26.20%	36.60%	32.00%	19.70%
6–7 times	77	24	14	110	169	6
	17.30%	8.30%	9.40%	26.60%	38.60%	4.40%
>7 times	45	33	12	54	66	4
	10.10%	11.50%	8.10%	13.10%	15.10%	2.90%
**Length**						
<15 min	36	9	8	19	13	8
	8.10%	3.10%	5.40%	4.60%	3.00%	5.80%
15–30 min	182	68	40	140	55	15
	41.00%	23.60%	26.80%	33.90%	12.60%	10.90%
30–60 min	159	98	55	195	173	34
	35.80%	34.00%	36.90%	47.20%	39.50%	24.80%
>60 min	67	113	46	59	197	80
	15.10%	39.20%	30.90%	14.30%	45.00%	58.40%
**Transportation mode**						
Walking (<5 min)	88	52	14	49	98	14
	19.80%	18.10%	9.40%	11.90%	22.40%	10.20%
Walking (5–10 min)	150	98	47	145	130	30
	33.80%	34.00%	31.50%	35.10%	29.70%	21.90%
Walking (10–20 min)	104	73	33	112	96	39
	23.40%	25.30%	22.10%	27.10%	21.90%	28.50%
Walking (20–30 min)	15	16	11	21	21	10
	3.40%	5.60%	7.40%	5.10%	4.80%	7.30%
Walking (>30 min)	30	9	7	35	44	6
	6.80%	3.10%	4.70%	8.50%	10.00%	4.40%
Public transport (<15 min)	10	8	7	14	12	5
	2.30%	2.80%	4.70%	3.40%	2.70%	3.60%
Public transport (15–30 min)	13	9	6	14	10	10
	2.90%	3.10%	4.00%	3.40%	2.30%	7.30%
Public transport (30–45 min)	15	13	16	13	15	14
	3.40%	4.50%	10.70%	3.10%	3.40%	10.20%
Public transport (45–60 min)	10	5	5	5	3	3
	2.30%	1.70%	3.40%	1.20%	0.70%	2.20%
Public transport (>60 min)	5	2	2	3	4	5
	1.10%	0.70%	1.30%	0.70%	0.90%	3.60%
Driving	3	1	0	0	1	0
	0.70%	0.30%	0.00%	0.00%	0.20%	0.00%
Others	1	2	1	1	4	1
	0.20%	0.70%	0.70%	0.20%	0.90%	0.70%
**Purpose of using community facility (multiple choices)**						
Physical exercise	3	37	6	10	299	0
	0.70%	12.80%	4.00%	2.40%	68.30%	0.00%
Learn new knowledge	4	79	49	7	6	29
	0.90%	27.40%	32.90%	1.70%	1.40%	21.20%
Social interaction	12	146	40	13	48	48
	2.70%	50.70%	26.80%	3.10%	11.00%	35.00%
Basic life needs	392	40	6	389	16	4
	88.30%	13.90%	4.00%	94.20%	3.70%	2.90%
Accompany family and friends	81	51	35	40	110	29
	18.20%	17.70%	23.50%	9.70%	25.10%	21.20%
Volunteer work	7	58	4	4	9	28
	1.60%	20.10%	2.70%	1.00%	2.10%	20.40%
Entertainment	45	66	48	9	195	26
	10.10%	22.90%	32.20%	2.20%	44.50%	19.00%
**Satisfaction level**						
Very dissatisfied	2	2	1	6	1	0
	0.50%	0.70%	0.70%	1.50%	0.20%	0.00%
Dissatisfied	16	9	10	31	17	1
	3.60%	3.10%	6.70%	7.50%	3.90%	0.70%
Fair	108	87	59	123	118	30
	24.30%	30.20%	39.60%	29.80%	26.90%	21.90%
Satisfied	280	171	73	227	254	82
	63.10%	59.40%	49.00%	55.00%	58.00%	59.90%
Very satisfied	38	19	6	26	48	24
	8.60%	6.60%	4.00%	6.30%	11.00%	17.50%

**Table 4:** Generalized estimating equation (GEE) models’ estimates for visitation frequency of community facilities.

	Commercial Facility				Community Service Facility				Cultural Facility				Municipal Facility				Leisure Facility				Religious Facility			
	OR	95% CI		Sig.	OR	95% CI		Sig.	OR	95% CI		Sig.	OR	95% CI		Sig.	OR	95% CI		Sig.	OR	95% CI		Sig.
**Distribution/Provision**																								
Number of community facilities	0.963	0.953	0.974	<0.001 **	0.886	0.774	1.013	0.077	1.075	0.835	1.384	0.574	0.824	0.699	0.973	0.022 *	0.988	0.967	1.009	0.260	1.018	0.998	1.040	0.084
Ratio of residents to facility	0.325	0.230	0.460	<0.001 **	0.835	0.628	1.111	0.217	0.978	0.833	1.148	0.784	0.958	0.885	1.037	0.287	0.907	0.740	1.112	0.349	1.121	0.952	1.319	0.171
**Perceived planning and design considerations**																								
Size	1.063	0.838	1.348	0.614	0.938	0.624	1.412	0.760	1.000	0.511	1.956	1.000	1.058	0.794	1.410	0.698	1.319	0.904	1.924	0.151	1.023	0.602	1.738	0.934
Adequate number	1.106	0.813	1.504	0.522	1.388	1.006	1.917	0.046 *	1.548	0.954	2.512	0.077	1.257	1.030	1.534	0.025 *	0.726	0.523	1.006	0.054	1.724	0.805	3.690	0.161
Diversity	0.787	0.562	1.100	0.161	0.721	0.496	1.046	0.085	1.425	0.825	2.462	0.204	1.147	0.848	1.552	0.374	0.959	0.667	1.379	0.820	1.185	0.558	2.516	0.658
Proximity to home	1.258	0.811	1.949	0.305	1.432	1.068	1.921	0.017 *	1.104	0.746	1.633	0.623	1.172	0.802	1.712	0.414	1.230	0.980	1.542	0.074	1.154	0.596	2.235	0.671
Proximity to public transport	1.238	0.915	1.674	0.166	0.897	0.623	1.292	0.561	1.199	0.762	1.886	0.432	1.242	0.837	1.843	0.281	1.239	0.982	1.563	0.071	1.165	0.558	2.433	0.684
Proximity to other amenities	1.255	1.033	1.525	0.022 *	1.436	1.070	1.927	0.016 *	0.880	0.543	1.427	0.606	0.928	0.767	1.121	0.437	1.119	0.796	1.571	0.518	0.597	0.270	1.317	0.201
**Purpose of using community facility**																								
Physical exercise	-	-	-	-	1.802	0.917	3.542	0.088	-	-	-	-	-	-	-	-	1.433	0.948	2.166	0.088	-	-	-	-
Acquire new knowledge	-	-	-	-	1.388	0.804	2.394	0.239	1.202	0.560	2.580	0.637	-	-	-	-	-	-	-	-	1.602	0.484	5.302	0.440
Social interaction	-	-	-	-	0.983	0.649	1.487	0.934	1.641	0.894	3.014	0.110	-	-	-	-	1.496	0.883	2.535	0.134	0.467	0.112	1.946	0.296
Basic life needs	0.646	0.358	1.164	0.146	2.058	0.953	4.445	0.066	-	-	-	-	3.081	1.676	5.663	<0.001 **	-	-	-	-	-	-	-	-
Accompany family and friends	1.337	0.908	1.969	0.141	4.116	1.491	11.363	0.006 **	4.124	1.652	10.297	0.002 **	1.771	0.882	3.556	0.108	1.618	1.073	2.440	0.022 *	5.688	1.591	20.334	0.007 **
Volunteer work	-	-	-	-	1.810	1.062	3.084	0.029 *	-	-	-	-	-	-		-	-	-	-	-	6.082	2.516	14.701	<0.001 **
Entertainment	1.184	0.721	1.945	0.505	1.178	0.671	2.068	0.569	0.976	0.495	1.923	0.944	-	-	-	-	1.142	0.694	1.877	0.601	0.882	0.281	2.764	0.829
**Socio-demographic characteristics**																								
Age	1.017	0.820	1.262	0.877	1.136	0.832	1.551	0.422	0.944	0.571	1.563	0.824	1.579	1.192	2.092	0.001 **	2.140	1.689	2.713	<0.001 **	0.478	0.335	0.684	<0.001 **
Gender																								
Male	0.729	0.544	0.977	0.034 *	0.897	0.595	1.351	0.603	1.423	0.858	2.360	0.172	0.523	0.311	0.877	0.014 *	0.743	0.565	0.978	0.034 *	2.174	0.976	4.839	0.057
Female	1.000	.	.	.	1.000	.	.	.	1.000	.	.	.	1.000	.	.	.	1.000	.	.	.	1.000	.	.	.
District																								
Sham Shui Po	0.724	0.543	0.964	0.027 *	0.241	0.099	0.585	0.002 *	0.747	0.336	1.658	0.473	0.233	0.049	1.107	0.067	0.610	0.332	1.122	0.112	0.420	0.209	0.845	0.015 *
Kwun Tong	1.000	.	.	.	1.000	.	.	.	1.000	.	.	.	1.000	.	.	.	1.000	.	.	.	1.000	.	.	.

Notes: CI = confidence interval; OR = odds ratio. * Correlation is significant at the 0.05 level (two-tailed). ** Correlation is significant at the 0.01 level (two-tailed).

**Table 5:** Generalized estimating equation (GEE) models’ estimates for length of stay in community facilities.

	Commercial Facility				Community Service Facility				Cultural Facility				Municipal Facility				Leisure Facility				Religious Facility			
	OR	95% CI		Sig.	OR	95% CI		Sig.	OR	95% CI		Sig.	OR	95% CI		Sig.	OR	95% CI		Sig.	OR	95% CI		Sig.
**Distribution/Provision**																								
Number of community facilities	0.947	0.928	0.966	<0.001 **	0.924	0.817	1.046	0.213	0.905	0.763	1.072	0.248	0.916	0.870	0.965	0.001 **	0.983	0.975	0.990	<0.001 **	1.006	0.981	1.032	0.624
Ratio of residents to facility	0.459	0.243	0.867	0.016 *	0.877	0.693	1.111	0.278	0.928	0.880	0.979	0.006 **	1.035	1.000	1.071	0.051	0.770	0.709	0.836	<0.001 **	0.969	0.867	1.083	0.573
**Perceived planning and design considerations**																								
Size	1.373	0.907	2.080	0.134	0.885	0.617	1.269	0.506	0.737	0.459	1.185	0.208	1.152	0.874	1.519	0.316	1.509	1.226	1.857	<0.001 **	0.943	0.498	1.784	0.856
Adequate number	1.554	1.135	2.126	0.006 **	0.986	0.687	1.415	0.941	0.961	0.624	1.480	0.856	1.005	0.702	1.440	0.978	0.934	0.685	1.273	0.666	1.118	0.637	1.961	0.698
Diversity	0.783	0.574	1.069	0.124	0.865	0.590	1.268	0.457	1.860	1.320	2.620	<0.001 **	1.046	0.787	1.391	0.755	0.683	0.511	0.915	0.010 *	0.481	0.287	0.808	0.006 **
Proximity to home	0.863	0.648	1.148	0.311	1.204	0.723	2.007	0.476	0.853	0.464	1.566	0.608	0.788	0.578	1.075	0.133	1.217	0.851	1.741	0.281	0.992	0.579	1.702	0.977
Proximity to public transport	0.881	0.670	1.158	0.364	1.824	1.331	2.501	<0.001 **	1.451	0.923	2.280	0.107	1.003	0.805	1.250	0.978	1.089	0.789	1.501	0.605	2.158	0.890	5.234	0.089
Proximity to other amenities	0.834	0.571	1.216	0.345	0.629	0.374	1.058	0.080	0.882	0.493	1.579	0.673	1.088	0.749	1.580	0.656	0.947	0.718	1.248	0.697	1.113	0.677	1.830	0.673
**Purpose of using community facility**																								
Physical exercise	-	-	-	-	1.526	0.732	3.181	0.259	-	-	-	-	-	-	-	-	2.565	1.806	3.643	<0.001 **	-	-	-	-
Acquire new knowledge	-	-	-	-	1.444	0.773	2.697	0.250	1.099	0.568	2.126	0.780	-	-	-	-	-	-	-	-	1.702	0.328	8.833	0.526
Social interaction	-	-	-	-	1.181	0.699	1.994	0.534	0.993	0.542	1.818	0.981	-	-	-	-	1.440	0.798	2.596	0.226	3.184	1.189	8.525	0.021 *
Basic life needs	0.729	0.346	1.535	0.405	0.355	0.182	0.693	0.002 **	-	-	-	-	0.805	0.449	1.444	0.468	-	-	-	-	-	-	-	-
Accompany family and friends	1.621	0.914	2.875	0.099	0.745	0.488	1.137	0.173	1.603	0.800	3.210	0.183	2.309	1.484	3.591	<0.001 **	1.540	1.056	2.246	0.025 *	0.690	0.191	2.494	0.571
Volunteer work	-	-	-	-	1.720	0.906	3.265	0.097	-	-	-	-	-	-	-	-	-	-	-	-	0.791	0.314	1.995	0.620
Entertainment	2.085	1.066	4.082	0.032 *	0.878	0.396	1.947	0.748	2.221	1.038	4.755	0.040 *	-	-	-	-	1.829	1.342	2.493	<0.001 **	1.239	0.478	3.207	0.659
**Socio-demographic characteristics**																								
Age	1.025	0.807	1.302	0.838	1.348	1.043	1.742	0.023 *	1.280	0.689	2.378	0.435	1.121	0.906	1.386	0.294	1.164	0.952	1.422	0.138	1.502	0.962	2.345	0.074
Gender																								
Male	1.020	0.695	1.499	0.918	0.626	0.399	0.982	0.042 *	1.436	0.775	2.662	0.251	0.874	0.580	1.316	0.518	1.303	0.985	1.725	0.064	0.440	0.282	0.687	<0.001 **
Female	1.000	.	.	.	1.000	.	.	.	1.000	.	.	.	1.000	.	.	.	1.000	.	.	.	1.000	.	.	.
District																								
Sham Shui Po	0.418	0.268	0.651	<0.001 **	0.519	0.255	1.057	0.071	0.542	0.272	1.081	0.082	1.240	0.941	1.634	0.126	0.984	0.668	1.450	0.935	2.658	1.099	6.432	0.030 *
Kwun Tong	1.000	.	.	.	1.000	.	.	.	1.000	.	.	.	1.000	.	.	.	1.000	.	.	.	1.000	.	.	.

Notes: CI = confidence interval; OR = odds ratio. * Correlation is significant at the 0.05 level (two-tailed). ** Correlation is significant at the 0.01 level (two-tailed).

**Table 6:** Results of generalized estimating equation (GEE) models’ estimates for satisfaction level with community facilities.

	Commercial Facility				Community Service Facility				Cultural Facility				Municipal Facility				Leisure Facility				Religious Facility			
	OR	95% CI		Sig.	OR	95% CI		Sig.	OR	95% CI		Sig.	OR	95% CI		Sig.	OR	95% CI		Sig.	OR	95% CI		Sig.
**Usage pattern**																								
Frequency	0.998	0.723	1.378	0.990	0.814	0.584	1.135	0.225	1.338	0.766	2.338	0.306	0.884	0.698	1.120	0.308	0.997	0.782	1.271	0.981	1.488	0.970	2.282	0.069
Length	1.038	0.798	1.349	0.782	1.078	0.830	1.402	0.573	1.344	0.811	2.226	0.251	1.259	0.951	1.668	0.108	1.301	0.965	1.754	0.084	1.111	0.742	1.664	0.609
**Distribution/Provision**																								
Number of community facilities	1.020	1.007	1.033	0.002 **	1.078	0.919	1.263	0.358	1.160	0.938	1.434	0.172	0.973	0.888	1.067	0.562	0.983	0.967	0.999	0.033 *	0.999	0.968	1.031	0.943
Ratio of residents to facility	2.097	1.256	3.502	0.005 **	1.555	1.196	2.022	0.001 *	1.070	0.998	1.148	0.056	0.962	0.910	1.016	0.164	0.912	0.825	1.010	0.076	0.989	0.847	1.156	0.893
**Perceived planning and design considerations**																								
Size	1.262	0.879	1.812	0.207	1.199	0.733	1.961	0.470	1.375	0.625	3.025	0.428	1.758	1.192	2.592	0.004 **	1.840	1.334	2.539	<0.001 **	1.499	0.726	3.095	0.273
Adequate number	1.090	0.687	1.728	0.715	1.484	0.978	2.250	0.063	1.497	0.773	2.900	0.232	0.906	0.658	1.248	0.546	1.239	0.893	1.717	0.200	0.468	0.219	0.998	0.049 *
Diversity	2.126	1.412	3.201	<0.001 **	1.321	0.754	2.316	0.331	0.906	0.511	1.606	0.735	1.957	1.430	2.678	<0.001 **	1.632	1.097	2.428	0.016 *	1.278	0.523	3.126	0.591
Proximity to home	1.203	0.887	1.631	0.235	1.379	0.746	2.550	0.305	1.342	0.763	2.361	0.307	1.047	0.824	1.329	0.708	1.289	1.019	1.630	0.035 *	1.701	0.691	4.189	0.248
Proximity to public transport	1.218	0.941	1.577	0.135	1.229	0.715	2.115	0.456	1.539	0.846	2.801	0.158	1.130	0.778	1.642	0.521	1.087	0.883	1.338	0.432	4.627	1.277	16.769	0.020 *
Proximity to other amenities	1.740	1.287	2.353	<0.001 **	1.503	0.928	2.435	0.098	3.371	1.808	6.284	<0.001 **	1.110	0.831	1.483	0.478	1.291	0.905	1.841	0.159	0.699	0.216	2.259	0.549
**Purpose of using community facility**																								
Physical exercise	-	-	-	-	4.699	1.121	19.699	0.034	-	-	-	-	-	-	-	-	1.166	0.679	2.005	0.578	-	-	-	-
Acquire new knowledge	-	-	-	-	0.790	0.502	1.245	0.310	1.071	0.461	2.487	0.874	-	-	-	-	-	-	-	-	2.736	1.161	6.444	0.021 *
Social interaction	-	-	-	-	1.096	0.673	1.782	0.713	1.610	0.510	5.086	0.417	-	-	-	-	1.018	0.473	2.191	0.964	0.268	0.086	0.831	0.023 *
Basic life needs	1.516	0.620	3.708	0.362	0.809	0.285	2.299	0.691	-	-	-	-	2.062	1.063	4.001	0.032 *	-	-	-	-	-	-	-	-
Accompany family and friends	1.741	0.966	3.139	0.065	0.391	0.194	0.785	0.008 **	0.798	0.347	1.839	0.597	0.731	0.338	1.581	0.425	0.710	0.400	1.261	0.242	0.829	0.247	2.780	0.762
Volunteer work	-	-	-	-	1.069	0.408	2.801	0.892	-	-	-	-	-	-	-	-	-	-	-	-	0.590	0.249	1.399	0.231
Entertainment	1.711	0.973	3.009	0.062	1.881	0.781	4.533	0.159	1.223	0.566	2.642	0.609	-	-	-	-	1.067	0.701	1.624	0.763	0.997	0.315	3.155	0.996
**Socio-demographic characteristics**																								
Age	1.134	0.839	1.533	0.412	1.691	1.066	2.681	0.026 *	0.956	0.500	1.828	0.891	1.218	0.850	1.745	0.282	1.092	0.811	1.470	0.563	1.780	1.063	2.981	0.028 *
Gender																								
Male	0.760	0.523	1.107	0.153	0.868	0.511	1.475	0.601	0.665	0.221	1.997	0.467	0.795	0.529	1.194	0.269	1.380	1.026	1.857	0.033 *	0.846	0.382	1.874	0.681
Female	1.000	.	.	.	1.000	.	.	.	1.000	.	.	.	1.000	.	.	.	1.000	.	.	.	1.000	.	.	.
District																								
Sham Shui Po	2.130	1.491	3.042	<0.001 **	4.534	1.673	12.290	0.003 **	1.788	0.862	3.708	0.118	0.924	0.562	1.521	0.757	0.899	0.553	1.461	0.667	4.045	0.953	17.175	0.058
Kwun Tong	1.000	.	.	.	1.000	.	.	.	1.000	.	.	.	1.000	.	.	.	1.000	.	.	.	1.000	.	.	.

Notes: CI = confidence interval; OR = odds ratio. * Correlation is significant at the 0.05 level (two-tailed). ** Correlation is significant at the 0.01 level (two-tailed).

**Table 7:** Results of generalized estimating equation (GEE) models’ estimates for physical well-being.

		Commercial Facility				Community Service Facility				Cultural Facility				Municipal Facility				Leisure Facility				Religious Facility			
		Coefficient	95% CI		Sig.	Coefficient	95% CI		Sig.	Coefficient	95% CI		Sig.	Coefficient	95% CI		Sig.	Coefficient	95% CI		Sig.	Coefficient	95% CI		Sig.
Intercept		2.694	2.075	3.314	<0.001 **	3.443	2.504	4.383	<0.001 **	3.007	2.100	3.914	<0.001 **	2.572	2.142	3.001	<0.001 **	3.598	2.856	4.339	<0.001 **	3.928	2.784	5.071	<0.001 **
**Usage pattern**																									
Frequency		0.143	0.040	0.246	0.007 **	0.131	0.020	0.242	0.021 *	0.056	−0.100	0.212	0.483	0.137	0.038	0.236	0.007 **	0.045	−0.083	0.174	0.489	0.250	0.121	0.378	<0.001 **
Length		0.009	−0.103	0.121	0.874	−0.061	−0.203	0.080	0.394	0.053	−0.117	0.224	0.539	0.098	0.008	0.189	0.034 *	0.028	−0.118	0.173	0.710	−0.075	−0.277	0.128	0.471
Satisfaction level		0.139	0.022	0.257	0.020 *	0.070	−0.075	0.214	0.343	0.127	−0.009	0.262	0.067	0.146	0.024	0.269	0.019 *	0.008	−0.160	0.177	0.924	−0.060	−0.246	0.127	0.531
**Distribution/Provision**																									
Number of community facilities		−0.001	−0.013	0.011	0.864	0.013	−0.051	0.076	0.691	−0.068	−0.132	−0.005	0.035 *	0.038	−0.020	0.095	0.200	−0.003	−0.016	0.011	0.705	−0.021	−0.031	−0.011	<0.001 **
Ratio of residents to facility		0.246	−0.119	0.611	0.186	−0.060	−0.211	0.090	0.431	0.015	−0.017	0.048	0.351	−0.014	−0.037	0.009	0.224	−0.017	−0.112	0.077	0.719	−0.059	−0.099	−0.019	0.004 **
**Socio-demographic characteristics**																									
Age		−0.252	−0.327	−0.177	<0.001 **	−0.227	−0.359	−0.094	0.001 **	−0.178	−0.324	−0.032	0.017 *	−0.228	−0.331	−0.125	<0.001 **	−0.255	−0.328	−0.183	<0.001 **	−0.164	−0.322	−0.007	0.040 *
Gender																									
	Male	0.184	0.029	0.339	0.020 *	0.154	−0.021	0.329	0.084	0.129	−0.082	0.341	0.231	0.145	−0.022	0.311	0.089	0.182	0.025	0.338	0.023 *	0.169	−0.020	0.359	0.080
	Female	0.000				0.000				0.000				0.000				0.000				0.000			
District																									
	Sham Shui Po	0.273	−0.027	0.573	0.074	0.282	−0.016	0.579	0.064	0.479	0.023	0.934	0.039 *	0.219	−0.108	0.546	0.190	0.174	−0.179	0.528	0.334	0.460	0.133	0.786	0.006 **
	Kwun Tong	0.000				0.000				0.000				0.000				0.000				0.000			
QIC		392.766				266.889				104.532				345.444				411.495				114.904			

Notes: CI = confidence interval; QIC = Quasi Likelihood under Independence Model Criterion. * Correlation is significant at the 0.05 level (two-tailed). ** Correlation is significant at the 0.01 level (two-tailed).

**Table 8:** Results of generalized estimating equation (GEE) models’ estimates for psychological well-being.

		Commercial Facility				Community Service Facility				Cultural Facility				Municipal Facility				Leisure Facility				Religious Facility			
		Coefficient	95% CI		Sig.	Coefficient	95% CI		Sig.	Coefficient	95% CI		Sig.	Coefficient	95% CI		Sig.	Coefficient	95% CI		Sig.	Coefficient	95% CI		Sig.
Intercept		−2.437	−3.108	−1.766	<0.001 **	−2.260	−3.450	−1.071	<0.001 **	−2.218	−3.320	−1.116	<0.001 **	−2.410	−2.963	−1.857	<0.001 **	−1.892	−2.754	−1.030	<0.001 **	−1.913	−3.163	−0.663	0.003 **
**Usage pattern**																									
Frequency		0.010	−0.071	0.090	0.811	0.104	−0.069	0.276	0.239	−0.111	−0.274	0.053	0.184	0.145	0.031	0.260	0.013 *	0.083	−0.049	0.215	0.219	−0.138	−0.491	0.215	0.444
Length		0.107	0.019	0.195	0.017 *	0.092	−0.052	0.237	0.210	0.150	0.037	0.264	0.009 **	0.081	−0.052	0.214	0.231	0.060	−0.048	0.168	0.274	0.339	0.187	0.491	<0.001 **
Satisfaction level		0.441	0.289	0.593	<0.001 **	0.394	0.210	0.579	<0.001 **	0.295	0.048	0.542	0.019 *	0.392	0.255	0.528	<0.001 **	0.338	0.176	0.500	<0.001 **	0.217	−0.103	0.537	0.183
**Distribution/Provision**																									
Number of community facilities		−0.007	−0.019	0.004	0.204	−0.058	−0.112	−0.003	0.039 *	0.003	−0.120	0.126	0.966	−0.028	−0.070	0.015	0.201	−0.005	−0.024	0.015	0.638	−0.009	−0.022	0.004	0.165
Ratio of residents to facility		0.043	−0.551	0.636	0.888	−0.029	−0.131	0.073	0.579	0.030	−0.016	0.077	0.198	0.009	−0.017	0.034	0.506	−0.095	−0.220	0.030	0.135	−0.058	−0.136	0.020	0.146
**Socio-demographic characteristics**																									
Age		0.193	0.083	0.304	0.001 **	0.245	0.090	0.400	0.002 **	0.199	−0.014	0.412	0.067	0.158	0.050	0.266	0.004 **	0.126	0.029	0.223	0.011 *	0.170	0.019	0.321	0.028 *
Gender																									
	Male	0.026	−0.133	0.186	0.746	−0.018	−0.189	0.153	0.839	−0.050	−0.310	0.211	0.707	0.055	−0.133	0.242	0.568	0.034	−0.133	0.200	0.690	−0.004	−0.318	0.311	0.981
	Female	0.000				0.000				0.000				0.000				0.000				0.000			
District																									
	Sham Shui Po	0.144	−0.295	0.583	0.521	−0.039	−0.445	0.367	0.850	0.359	−0.160	0.878	0.175	0.080	−0.244	0.404	0.627	0.143	−0.255	0.541	0.482	−0.119	−0.540	0.301	0.578
	Kwun Tong	0.000				0.000				0.000				0.000				0.000				0.000			
QIC		408.757				258.258				146.607				356.524				395.369				140.599			

Notes: CI = confidence interval; QIC = Quasi Likelihood under Independence Model Criterion. * Correlation is significant at the 0.05 level (two-tailed). ** Correlation is significant at the 0.01 level (two-tailed).

## Data Availability

Data available on request due to restrictions, e.g., privacy or ethical.

## Supplementary Materials

The following supporting information can be downloaded at: https://www.mdpi.com/article/10.3390/ijerph191610297/s1, File S1: Questionnaire sample.

## Appendix A

**Table A1:** Measurement of physical and psychological well-being.

**Physical well-being**	‘Can you walk 360 m? (one sports field)’	0 (Unable); 1/3 (Can do it with help); 2/3 (Some difficulty); 1 (No difficulty)
	‘Do you have any chronic illness(es)?’	0 (Yes); 1 (No)
	‘Do you have any illness that limits your social activities participation?’	
	‘Do you have any illness that limits your ability to take care of yourself?’	
**Psychological well-being**	‘In the last week, I have felt cheerful and have been in good spirits.’	1 (strongly disagree) to 5 (strongly agree)
	‘In the last week, I have felt calm and relaxed.’	
	‘In the last week, I have felt active and vigorous.’	
	‘In the last week, I have woken up feeling fresh and well rested.’	
	‘In the last week, my daily life has been filled with things that interest me.’	

Notes: The variable ‘Physical well-being’ is a transformation of the original measures, as shown in the table; each question has a full score of one. The variable ‘Psychological well-being’ is a combination of five items shown in the table by using factor analysis. Reference: He et al. (2020) [68].
