# Supplementary material for: Older People’s Usage Pattern, Satisfaction with Community Facility and Well-Being in Urban Old Districts
Source: Int J Environ Res Public Health. 2022 Aug 18;19(16):10297. doi: 10.3390/ijerph191610297 (PMC9408318; doi:10.3390/ijerph191610297)
Supplement: Supplementary file 1 [file ijerph-19-10297-s001.zip › ijerph-1854956-supplementary.pdf]

File S1: Questionnaire sample

PART I Usage pattern of community facilities

1. Please indicate the frequency of visiting following community facilities in the past week：

	A (0)	B (1-2)	C (3-5)	D (6-7)	E (>7)
Commercial facility					
Community service facility					
Cultural facility					
Municipal facility					
Leisure facility					
Religious facility					

2. Please indicate the time period per visit of following community facilities in the past week：

	A (<15 min)	B (15-30 min)	C (30-60 min)	D (>60 min)
Commercial facility				
Community service facility				
Cultural facility				
Municipal facility				
Leisure facility				
Religious facility				

3. Please indicate your purpose of visiting following community facilities（multi-choices）：

	Physical exercise	Learn new knowledge	Social interaction	Basic life needs	Accompany with family and friends	Volunteer work	Entertainment	Others
Commercial facility								

Community service facility								
Cultural facility								
Municipal facility								
Leisure facility								
Religious facility								

4. Please indicate your transportation mode of visiting following community facilities.

	Public transport					Walk					Car	others
	<15 min	15-30 min	30-45 min	45-60 min	>60 min	<5 min	5-10 min	10-20 min	20-30 min	>30 min		
Commercial facility												
Community service facility												
Cultural facility												
Municipal facility												
Leisure facility												
Religious facility												

5. Please indicate your satisfaction level with following community facilities.

	Very dissatisfied	Dissatisfied	Fair	Satisfied	Very satisfied
Commercial facility					
Community service facility					
Cultural facility					
Municipal facility					
Leisure facility					
Religious facility					

PART II Planning and design of community facilities

Please rate your agreement with the following statements.

（1. Strongly disagree；2. Disagree；3.Fair；4.Agree；5. Strongly agree）

1	The size (space) of the community facilities is enough in my neighborhood.					
	Commercial facility	1	2	3	4	5
	Community service facility	1	2	3	4	5
	Cultural facility	1	2	3	4	5
	Municipal facility	1	2	3	4	5
	Leisure facility	1	2	3	4	5
	Religious facility	1	2	3	4	5
2	The number of community facilities is adequate in my neighborhood.					
	Commercial facility	1	2	3	4	5
	Community service facility	1	2	3	4	5
	Cultural facility	1	2	3	4	5
	Municipal facility	1	2	3	4	5
	Leisure facility	1	2	3	4	5
	Religious facility	1	2	3	4	5
3	The community facilities are diversity in my neighborhood.					
	Commercial facility	1	2	3	4	5
	Community service facility	1	2	3	4	5
	Cultural facility	1	2	3	4	5
	Municipal facility	1	2	3	4	5
	Leisure facility	1	2	3	4	5
	Religious facility	1	2	3	4	5

4	The community facilities are proximity to my home.					
	Commercial facility	1	2	3	4	5
	Community service facility	1	2	3	4	5
	Cultural facility	1	2	3	4	5
	Municipal facility	1	2	3	4	5
	Leisure facility	1	2	3	4	5
	Religious facility	1	2	3	4	5
5	The community facilities are proximity to public transport stations.					
	Commercial facility	1	2	3	4	5
	Community service facility	1	2	3	4	5
	Cultural facility	1	2	3	4	5
	Municipal facility	1	2	3	4	5
	Leisure facility	1	2	3	4	5
	Religious facility	1	2	3	4	5
6	The community facilities are proximity to other amenities.					
	Commercial facility	1	2	3	4	5
	Community service facility	1	2	3	4	5
	Cultural facility	1	2	3	4	5
	Municipal facility	1	2	3	4	5
	Leisure facility	1	2	3	4	5
	Religious facility	1	2	3	4	5

PART III Well-being

Physical well-being

1. Can you walk 360 m? (one sports field)

○ No difficulty ○ Some difficulty ○ Can do it with help ○ Unable

2. Do you have any chronic illness(es)?

○ Yes ○ No

3. Do you have any illness that limits your social activities participation?

○ Yes ○ No

4. Do you have any illness that limits your ability to take care of yourself?

○ Yes ○ No

Psychological well-being

（1. Strongly disagree；2. Disagree；3.Fair；4.Agree；5. Strongly agree）

1	In the last one week, I have felt cheerful and have been in good spirits.	1	2	3	4	5
2	In the last one week, I have felt calm and relaxed.	1	2	3	4	5
3	In the last one week, I have felt active and vigorous.	1	2	3	4	5
4	In the last one week, I have woken up feeling fresh and well-rested.	1	2	3	4	5
5	In the last one week, my daily life has been filled with things that interest me.	1	2	3	4	5

PART IV Personal information

Gender：○Male ○Female

Age：○55-60 ○61-70 ○71-80 ○81-90 ○>90

Education：○Primary school or lower ○Secondary school
○Post-secondary ○Graduate school or higher

Which TPU you lived in?

○255 ○260 ○261 ○262 ○263 ○264 ○265 ○266 ○267
○268 ○269 ○280 ○290 ○291 ○292 ○293 ○294 ○297
○298
